# The Sensory Histidine Kinase CusS of Escherichia coli Senses Periplasmic Copper Ions

**DOI:** 10.1128/spectrum.00291-23

**Published:** 2023-03-14

**Authors:** Jeanine Rismondo, Cornelia Große, Dietrich H. Nies

**Affiliations:** a Institute for Biology/Microbiology, Molecular Microbiology, Martin-Luther-University Halle-Wittenberg, Halle, Germany; South China Sea Institute Of Oceanology

**Keywords:** two-component regulatory systems, histidine kinases, copper resistance

## Abstract

Two-component regulatory systems composed of a membrane-bound sensor/sensory histidine kinase (HK) and a cytoplasmic, DNA-binding response regulator (RR) are often associated with transenvelope efflux systems, which export transition metal cations from the periplasm directly out of the cell. Although much work has been done in this field, more evidence is needed for the hypothesis that the respective two-component regulatory systems are indeed sensing periplasmic ions. If so, a regulatory circuit between the concentration of periplasmic metal cations, sensing of these metals, and control of expression of the genes for transenvelope efflux systems that remove periplasmic cations can be assumed. Escherichia coli possesses only one transenvelope efflux system for metal cations, the Cus system for export of Cu(I) and Ag(I). It is composed of the transenvelope efflux system CusCBA, the periplasmic copper chaperone CusF, and the two-component regulatory system CusS (HK) and CusR (RR). Using *phoA*- and *lacZ*-reporter gene fusions, it was verified that an assumed periplasmic part of CusS is located in the periplasm. CusS was more important for copper resistance in E. coli under anaerobic conditions than under aerobic conditions and in complex medium more than in mineral salts medium. Predicted copper-binding sites in the periplasmic part of CusS were identified that, individually, were not essential for copper resistance but were in combination. In summary, evidence was obtained that the two-component regulatory system CusSR that controls expression of *cusF* and *cusCBA* does indeed sense periplasmic copper ions.

**IMPORTANCE** Homeostasis of essential-but-toxic transition metal cations such as Zn(II) and Cu(II)/Cu(I) is an important contributor to the fitness of environmental bacteria and pathogenic bacteria during their confrontation with an infected host. Highly efficient removal of threatening concentrations of these metals can be achieved by the combined actions of an inner membrane with a transenvelope efflux system, which removes periplasmic ions after their export from the cytoplasm to this compartment. To understand the resulting metal cation homeostasis in the periplasm, it is important to know if a regulatory circuit exists between periplasmic metal cations, their sensing, and the subsequent control of the expression of the transenvelope efflux system. This publication adds evidence to the hypothesis that two-component regulatory systems in control of the expression of genes for transenvelope efflux systems do indeed sense metal cations in the periplasm.

## INTRODUCTION

Two-component regulatory systems are usually composed of a membrane-bound sensory histidine kinase (HK) and a cytoplasmic DNA-binding response regulator (RR), which is phosphorylated following a signal being sensed by the HK (1). The RR dimerizes within seconds after the HK has received the stimulus and activates transcription (2), for instance, of a reporter gene. In Escherichia coli, the two-component regulatory system CusRS is composed of the HK CusS and the RR CusR. It controls expression of the *cusCFBA* determinant for the Cu(I)- and Ag(I)-specific CusCBA transenvelope efflux system and a small periplasmic protein CusF, which together are responsible for copper and silver resistance in this bacterium (3–8). Ag(I), Cu(I), and also Cu(II) cations bind to a peptide representing the periplasmic domain of CusS and induce a conformational change (8, 9).

Metal ion-transporting transenvelope complexes, such as CusCBA, are important contributors to metal resistance in Gram-negative bacteria (10). Transport is driven by the proton motive force. The “A” subunit of these transenvelope protein complexes, a member of the resistance, nodulation, and cell division (RND) superfamily (11), is located in the inner membrane and is responsible for the energetic coupling between proton import and cation export (12). CusA from E. coli and CzcA from Cupriavidus metallidurans are able to transport metal cations across a membrane after *in vitro* reconstitution (12–14).

In contrast to the *in vitro* data, *in vivo* evidence suggests that transport by CusCBA is mainly from the periplasm to outside the cell and not from the cytoplasm. In addition to the *cusCFBA* operon encoding the transmembrane metal efflux system CusCBA and the periplasmic Cu(I)-binding protein CusF, copper and silver resistance in Escherichia coli is mediated by the P_IB1_-type ATPase CopA and the periplasmic Cu(I) oxidase CueO, both of which are under the control of the cytoplasmic MerR-type regulator CueR, (3, 6, 7, 15–18). Deletion of *cueO* or of *cusCFBA* in a Δ*copA* background has only a small (or no) effect on copper resistance in E. coli, but deletion of *cueO* and *cusCFBA* in Δ*copA* leads to a nearly 2-fold decrease in copper resistance (19, 20). Consequently, Cus was not able to substitute for a missing CopA P_IB1_-type ATPase, which exports cytoplasmic Cu(I) to the periplasm, but was able to substitute a missing periplasmic Cu(I) oxidase. Because CueO oxidizes Cu(I) to Cu(II) to decrease the periplasmic concentration of the Cu(I) species, which is more toxic than Cu(II) (21, 22), but CusCBA is no Cu(I) oxidase, this indicates strongly that CusCBA exports periplasmic Cu(I) rather than cytoplasmic Cu(I). Under anaerobic conditions, Cus is more useful than CueO for copper resistance in E. coli because molecular oxygen is not available here as an electron acceptor to CueO-mediated Cu(I) oxidation (5, 21).

Cus is the only metal-exporting transenvelope efflux system in E. coli and is under control of the two-component regulatory system CusRS. Signaling and RR phosphorylation has been elucidated for CusS and related proteins. The structure of the kinase core domain of CusS, which is located in the cytoplasm, has been solved (23). A polypeptide predicted to constitute the periplasmic domain of CusS binds Ag(I) ions, but it has not been verified that this domain is located in the periplasm (8, 24). Moreover, it has also not been demonstrated that the assumed periplasmic domain of CusS is indeed in the periplasm. Should this be the case, this would shed some light on metal resistance of many bacteria and answer the question of whether a particular resistance system primarily counters the influence of periplasmic or cytoplasmic metal ions. If the first instance proved to be correct, then such a system would be under the control of a two-component regulatory system; in the second instance, control through a MerR-type one-component regulatory system would be expected. We provide evidence here that the HK CusS from E. coli indeed senses periplasmic copper ions.

## RESULTS

### CusS of E. coli contains a periplasmic domain.

**FIG 1:**
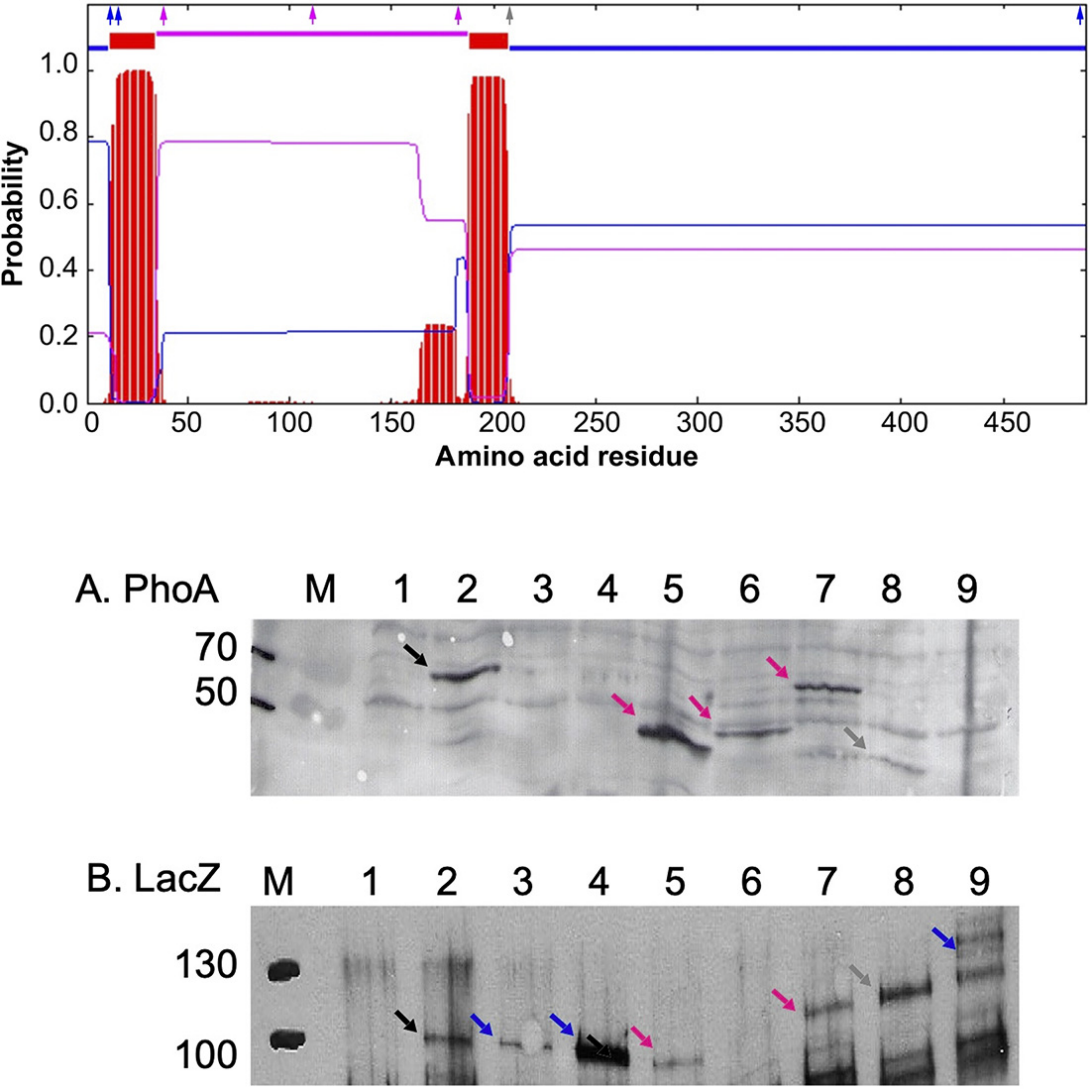
Topology of CusS from E. coli and CusS PhoA and LacZ fusion proteins. Top, a prediction of transmembrane alpha-helices is shown using TMHMM (25). Two clearly predicted transmembrane regions (red) are separated by a periplasmic domain (magenta) from amino acid position 35 to 187. Predicted cytoplasmic domains at the amino terminus and from position 208 to the carboxy terminus are labeled in blue. The small arrows give the LacZ and PhoA fusion sites, with the outcome cytoplasmic (blue), periplasmic (magenta), or unclear (gray). (A and B) The translationally fused genes were expressed in E. coli, crude extracts were prepared from 25 μg of dry mass per lane, and the proteins were analyzed by Western blotting using PhoA- or LacZ-specific polyclonal antibodies. PhoA fusions (A) and LacZ fusions (B) are shown. Size markers are Precision Plus Protein Western C Standards (kDa; M). The following are the lane designations: negative control (lane 1), positive control (lane 2), and *cusS-phoA* fusions at positions 15 (lane 3), 17 (lane 4), 38 (lane 5), 112 (lane 6), 185 (lane 7), 207 (lane 8), and 480 (lane 9). Signals are labeled by arrows in colors as shown on the top; controls are in black.

**FIG 2:**
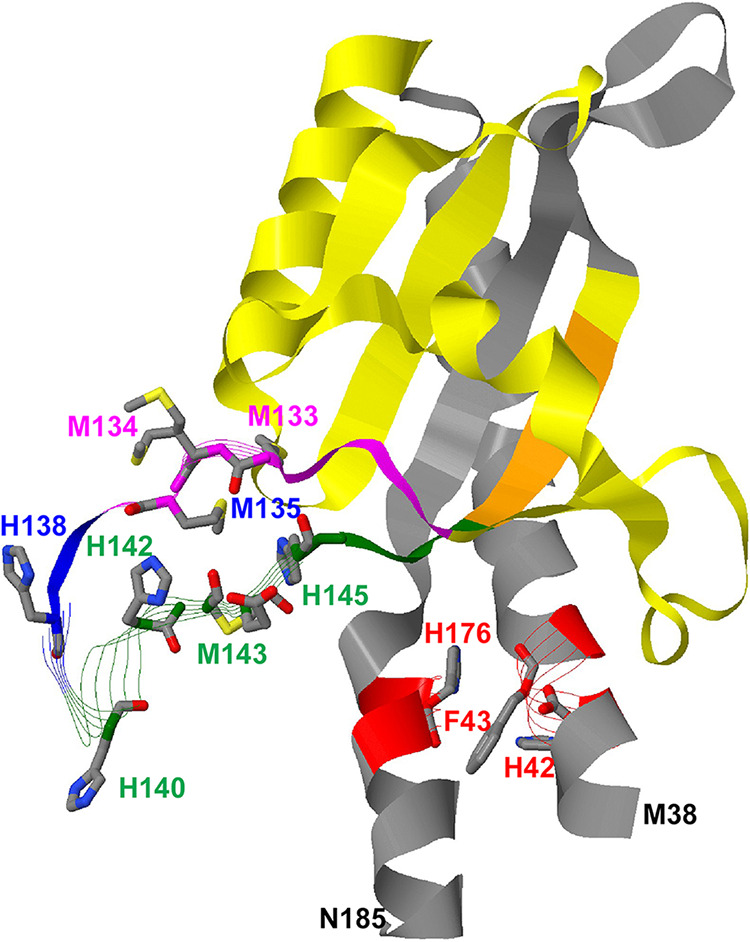
Structure of the periplasmic domain of CusS. The structure of the periplasmic domain from M38 to N185 is displayed as in Protein Data Bank ID 5KU5 (24). The positions of sites deleted in mutant proteins are shown: *cusS*Δ(129 to 138) in magenta and blue; *cusS*Δ(136 to 148) in blue and green; the overlapping positions 136, 137, and 138 in blue; positions 129 to 152 in *cusS*Δ(129 to 152) in orange; and everything else deleted in *cusS*Δ(67 to 153) in yellow. In the region of positions 129 to 148, the imidazole rings of histidine residues and the sulfur-containing methionine residues are in blue for nitrogen and yellow for sulfur atoms, respectively. These sites are not conserved in all CusS homologs. Please note that the M134 side chain has two possible positions, indicating high flexibility of this amino acyl residue. The residues in the dimer interphase that are involved in sensing are shown in red.

A TMHMM-based modeling (25) of CusS predicts that a periplasmic domain is present in CusS between amino acid positions 35 and 187 (Fig. 1). The structure of this domain had been solved (24) (Fig. 2). For an experimental verification of the prediction, C-terminal PhoA and LacZ protein fusions were constructed at several positions within the CusS polypeptide. The plasmids carrying the genes encoding these fusion proteins were introduced into E. coli, and the resulting alkaline phosphatase or beta-galactosidase enzyme activities were determined (Table 1). Alkaline phosphatase is only active in the periplasm, while beta-galactosidase can only function properly in the cytoplasm (26). Low alkaline phosphatase combined with high beta-galactosidase activities indicated a cytoplasmic location of the amino- and large carboxy-terminal part of CusS. In contrast, the measured high PhoA and low LacZ enzyme activities provided evidence for a periplasmic location of CusS when fusions were made at positions 38, 112, and 185 within the protein. High activity of both PhoA and LacZ was measured when the fusion was made at position 207, following the second predicted transmembrane alpha helix of CusS (Fig. 1, gray arrow).

**TABLE 1:** Topology of CusS

Position^*a*^	PhoA	LacZ	*Q* (LacZ/PhoA)
15	0.24 ± 0.01	24.7 ± 2.1	103
17	0.15 ± 0.07	40.1 ± 7.6	267
38	8.49 ± 0.62	16.4 ± 3.0	1.93
112	6.15 ± 0.87	8.98 ± 0.88	1.46
185	8.01 ± 0.39	22.6 ± 2.7	2.82
207	7.23 ± 0.40	40.4 ± 6.9	5.59
480	1.62 ± 0.72	63.0 ± 7.8	38.9

a Translational fusions of the *cusS* gene with the *phoA* and the *lacZ* genes were created at the indicated codon/amino acid residue positions as shown in Fig. 1. The specific activities of the respective fusion constructs were determined. The ratio Q between the LacZ and the PhoA activities indicated a cytoplasmic location of the PhoA and LacZ domains at the amino and carboxy termini (blue arrows in Fig. 1), a periplasmic location for those in light gray rows (magenta arrows in Fig. 1), and an ambiguous value for position 207 following the second transmembrane alpha-helix (dark gray row and gray arrow in Fig. 1).

Western blotting of the fusion proteins produced in E. coli cells after separation of polypeptides in crude extracts by SDS-PAGE (Fig. 1) yielded signals for LacZ fusion proteins at positions 15 and 17 (blue arrows) but not PhoA fusion proteins, which agreed with the low PhoA-specific activity detected (Table 1). At positions 38, 112, and 185, PhoA fusion proteins, but also some LacZ fusion proteins, could be visualized (magenta arrows). At position 207 (gray arrows), the LacZ fusion protein was clearly visible, while the corresponding PhoA activity showed degradation. Finally, at position 480, no PhoA fusion protein was detectable, but a LacZ fusion protein and many of its degradation products could be detected. The specific activities of the corresponding enzymes (Table 1) in combination with the presence or absence of the fusion proteins in E. coli (Fig. 1) clearly verified the predicted topology of CusS with a short cytoplasmic amino-terminal region and a large carboxy-terminal domain of CusS, two transmembrane alpha-helices, and a periplasmic domain between positions 35 and 187.

The structure of the periplasmic domain between positions 38 and 185 has been solved (24), and amino acid residues involved in binding of the Cu(I) proxy Ag(I) have been identified, namely, F43, H42, and H176 between alpha-helices leading down to the cytoplasmic membrane as well as S84, M133, M135, and H145 in a disordered loop of the periplasmic domain. The next question was whether the predicted metal-binding sites in the periplasmic domain of CusS (Fig. 2) were required for activation of *cus* via the HK CusS and the RR CusR.

### Sensing of copper by the periplasmic part of CusS is required for Cus-mediated copper resistance under anaerobic conditions.

**FIG 3:**
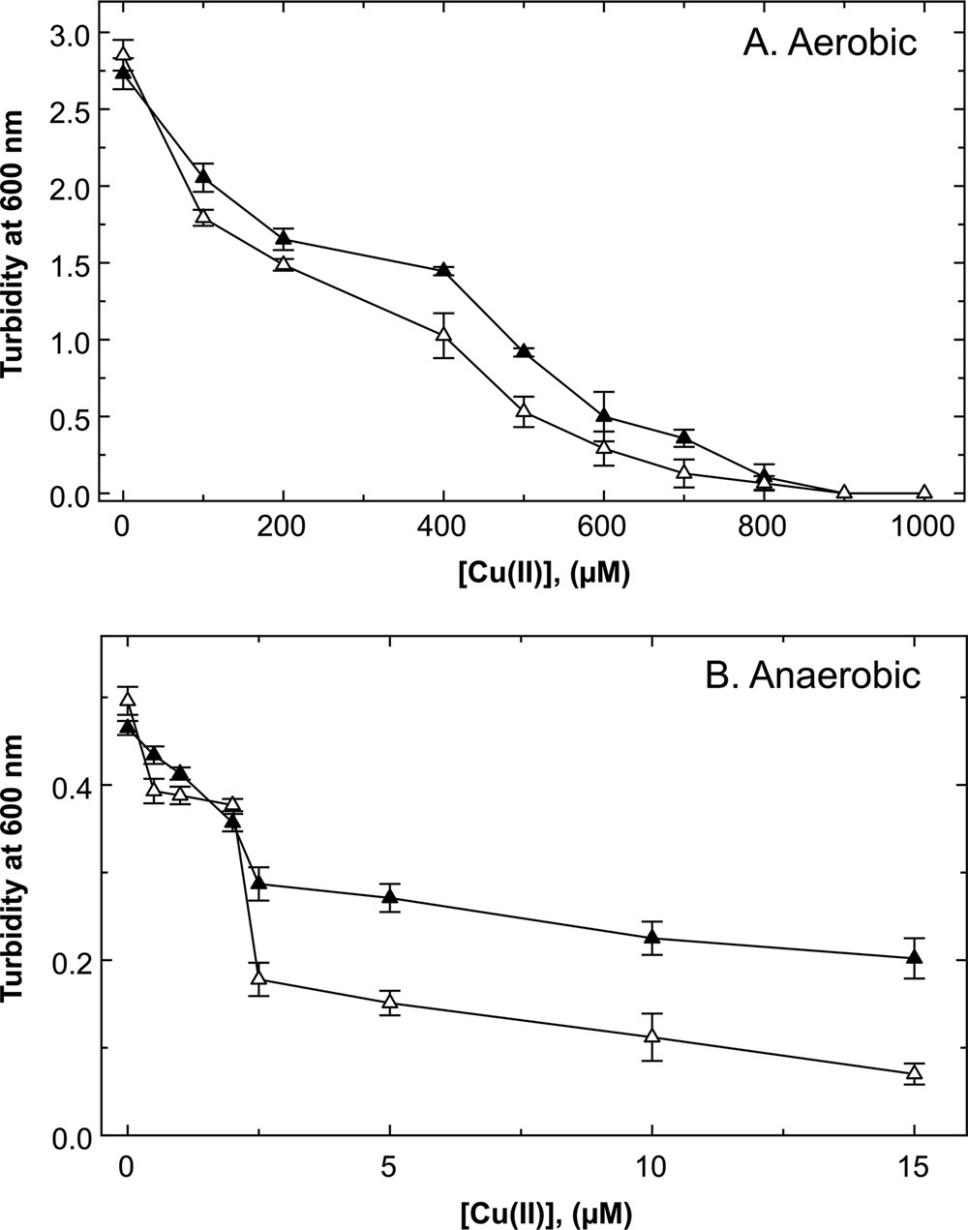
Effect of a Δ*cusS* deletion on copper resistance in E. coli Δ*copA ΔcueO* in mineral salts medium. (A and B) The *cusS* gene was deleted in the E. coli mutant strain Δ*copA ΔcueO*, and copper resistance was determined by dose-response experiments under aerobic (A; 6 h at 37°C with shaking) or anaerobic (B; 6 h at 37°C in Hungate tubes) conditions in TMM adapted for E. coli. Turbidity was determined at 600 nm. Strains Δ*copA ΔcueO* (closed triangles) and Δ*copA ΔcueO ΔcusS* (open triangles) are shown. Error bars represent standard deviation; *n* ≥ 3.

To investigate the contribution of the periplasmic part of CusS to copper resistance in E. coli, growth conditions had to be identified that provided a difference between a Δ*cusS* strain and its respective parent. In a Δ*copA* Δ*cueO* double mutant, the Cus system was the major remaining copper resistance system in E. coli. In Tris-buffered mineral salts medium (TMM) (Fig. 3), deletion of *cusS* had a clear effect under aerobic and anaerobic conditions. Under aerobic conditions, the 50% inhibitory concentration (IC_50_) of the triple mutant was about 250 μM Cu(II), and that of the double mutant was approximately 450 μM (Fig. 3A). Copper resistance under anaerobic conditions was significantly lower (Fig. 3B), with a difference between Δ*cusS* and its parent at copper concentrations above 2 μM. However, the differences between the triple mutant and the double mutant with an intact *cusS* gene were small in the Δ*copA ΔcueO* background, even in mineral salts medium, probably due to the strong decrease in copper resistance as result of the Δ*copA* deletion. Nevertheless, these data confirmed that Cus contributed to copper resistance, even under aerobic conditions when CueO is absent (5, 21).

**FIG 4:**
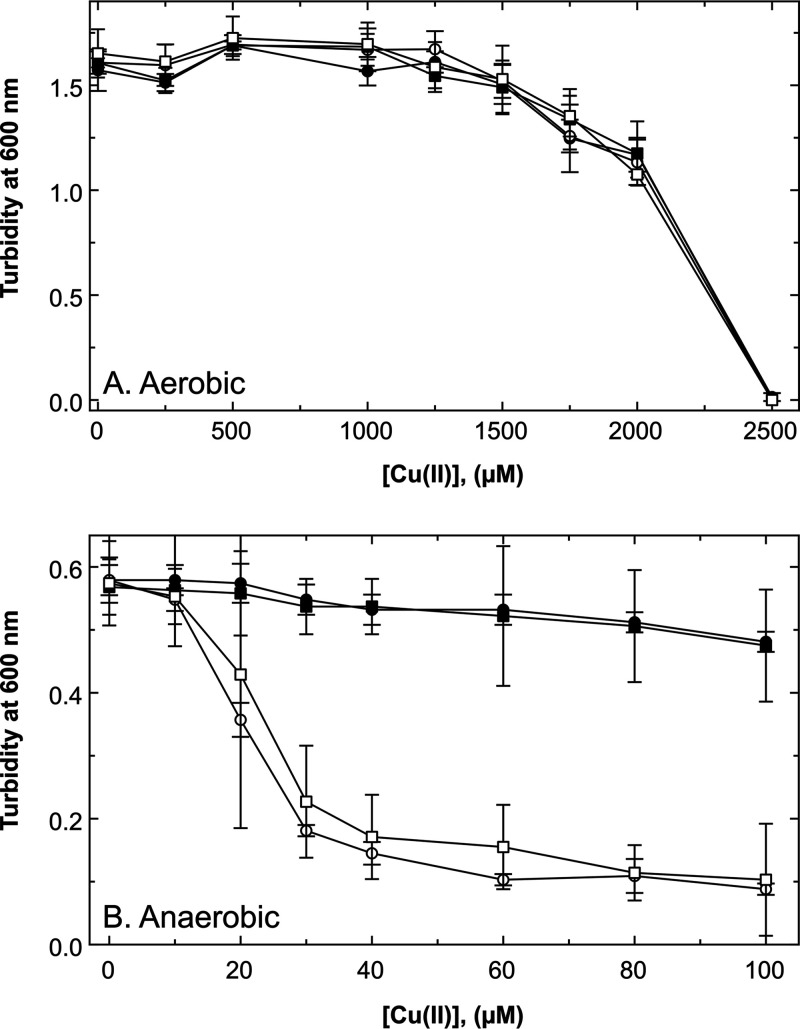
Effect of a Δ*cusS* deletion on copper resistance in E. coli in complex medium. The *cusS* gene was deleted in E. coli strain W3110 and its Δ*barA ΔuhpB ΔyedV* triple mutant, and copper resistance was determined by dose-response experiments under aerobic (A; 6 h at 37°C with shaking) or anaerobic (B; 6 h at 37°C in Hungate tubes) conditions in LB medium. Turbidity was determined at 600 nm. Parent strain W3110 (closed circles), Δ*cusS* (open circles), Δ*barA ΔuhpB ΔyedV* triple mutant (closed squares), and Δ*barA ΔuhpB ΔyedV ΔcusS* quadruple mutant (open squares) are shown. Error bars represent standard deviation; *n* ≥ 3.

In the complex medium LB, the IC_50_ of the wild-type strain under aerobic conditions was greater than 2 mM (Fig. 4). Under anaerobic conditions, the IC_50_ of the wild type was above 100 μM, while the IC_50_ of the Δ*cusS* single mutant was about 25 μM, revealing a sufficiently large difference between the presence and absence of *cusS* for the subsequent experiments; it also confirmed the important role of Cus under anaerobic conditions (5, 21).

Three HKs from E. coli were reported to cross-talk with Cus: BarA, UhpB, and YedV (27). The respective genes for these histidine kinases were deleted in E. coli and additionally *cusS* in the resulting triple mutant. Under aerobic conditions in LB, the copper resistance of a Δ*barA ΔuhpB ΔyedV* triple mutant, a Δ*barA ΔuhpB ΔyedV ΔcusS* quadruple mutant, a Δ*cusS* single mutant, and the parental strain was not significantly different (Fig. 4A). Under anaerobic conditions, copper resistance of the Δ*barA ΔuhpB ΔyedV* triple mutant was similar to that of the parental strain, while that of the Δ*barA ΔuhpB ΔyedV ΔcusS* quadruple mutant was similar to that of the Δ*cusS* single mutant (Fig. 4B). Deletion of *cusS* resulted in loss of copper resistance under anaerobic conditions, and the HKs BarA, UhpB, and YedV did not interfere with Cus-mediated copper resistance. Therefore, cross-talk between nonmetal-sensing HKs (27) was not evident in the control of Cus-mediated metal resistance.

**FIG 5:**
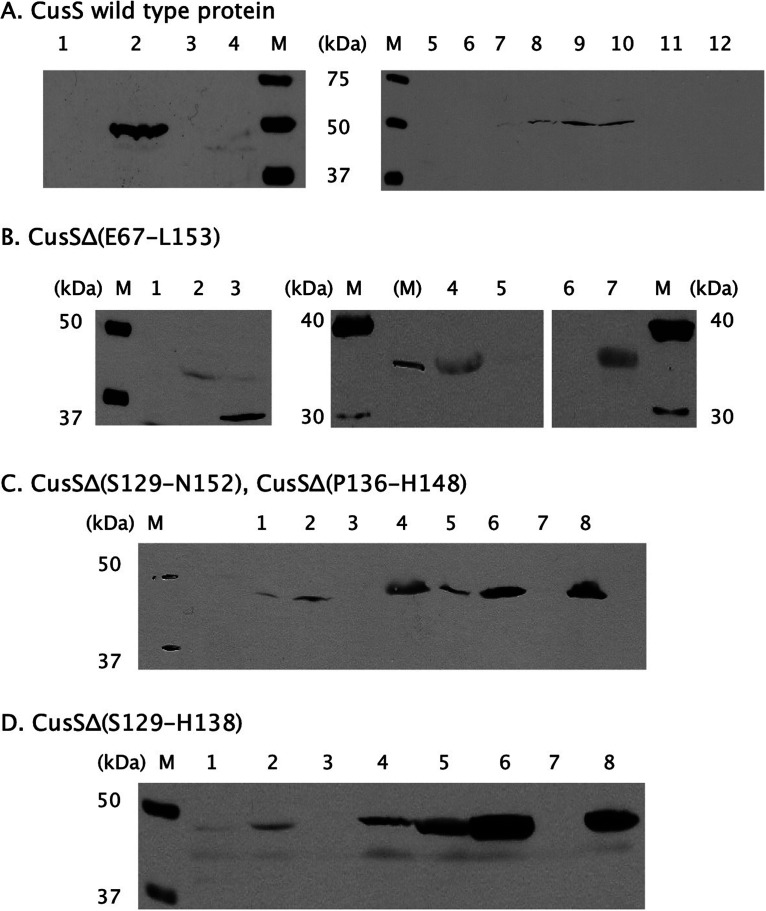
Production of CusS and mutant derivatives. Derivatives of the *cusS* genes were cloned in vector plasmid pASK-IBA7 as fusion proteins with an amino-terminal *Strep* tag II. The corresponding genes were expressed in E. coli strain BL21(DE3) after induction of gene expression with 10 μg/L or 200 μg/L anhydrotetracyclin (AHT). Fusion proteins were visualized by Western blotting with anti-*Strep*-Tactin horseradish peroxidase. In all blots, the Precision Plus Protein Western C Standards size marker (in kDa; M) is shown. (A) Wild-type CusS before induction (1), after addition of 200 μg/L AHT (2), vector controls without (3) and with AHT (4), crude extract (5), cell debris (7), membrane fraction (9), soluble cytoplasmic fraction (11) without AHT and after treatment with 10 μg/L AHT (6, 8, 10, and 12, respectively). (B) CusSΔ(E67 to L153) before induction (1), after treatment with 10 μg/L (2) or 200 μg/L (3) AHT, cell debris (4), crude extract (5), soluble cytoplasmic fraction (6), and membrane fraction (7) after induction with 10 μg/L AHT. (C) CusSΔ(S129 to N152) after treatment with 10 μg/L AHT, crude extract (1), cell debris (2), soluble fraction (3), and membrane fraction (4); lanes 5 to 8 are as for lanes 1 to 4 but for CusSΔ(P136 to W148). (D) CusSΔ(S129 to H148) after treatment with 10 μg/L AHT, crude extract (1), cell debris (2), soluble fraction (3), and membrane fraction (4); lanes 5 to 8 are as for lanes 1 to 4 but after treatment with 200 μg/L AHT.

**FIG 6:**
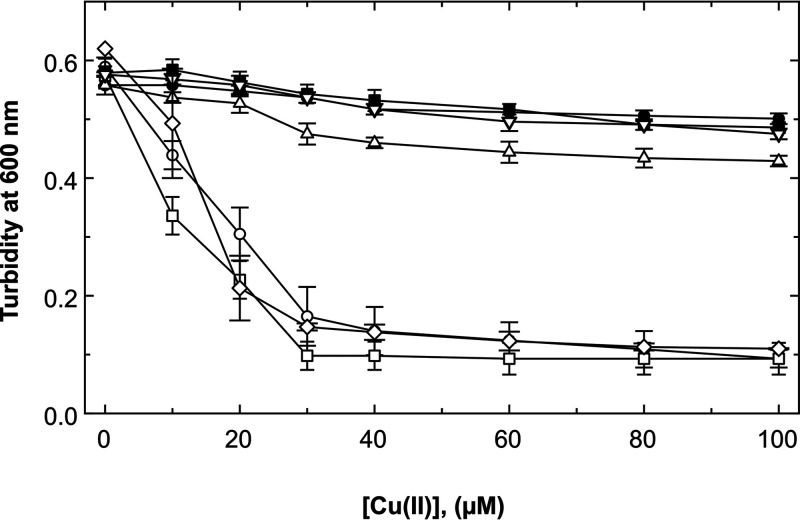
Copper resistance of Δ*cusS* mutants complemented in *trans*. The *cusS* gene was deleted in E. coli strain W3110, and the mutant strain was complemented in *trans* with *cusS* derivatives cloned in vector plasmid pASK-IBA7 encoding fusion proteins with an amino-terminal *Strep* Tag II. Copper resistance was determined by dose-response experiments under anaerobic conditions (6 h at 37°C in Hungate tubes) after growth in LB medium. Expression of the *cusS* gene was induced with 10 μg/L anhydrotetracycline. Turbidity was determined at 600 nm. Wild-type strain W3110(pASK-IB7) (closed circles,) and Δ*cusS* (pASK-IBA7) (open circles) are shown. Complemented strains contained as insertion into plasmid pASK-IBA7 with the genes for the CusS wild-type protein (pECD1169, closed squares), CusS(ΔE67 to L153, pECD1170, open diamonds), CusS(ΔS129 to N152, pECD1171, open squares), CusS(ΔS129 to H138, pECD1172, open inverted triangles), or CusS(ΔP136 to W148, pECD1173, open triangle). Error bars represent standard deviation; *n* ≥ 3.

CusS and mutant derivatives thereof were all produced in E. coli as *Strep*-tagged proteins from a plasmid carrying the cognate gene, so that the presence of the proteins in the cells and their location in the cytoplasmic membrane could be verified by western blotting. A CusS-specific signal was observed in the Western blot but was absent in the vector control (Fig. 5A). Even without the inducer anhydrotetracycline (AHT) being added, CusS could be detected in the membrane fraction (Fig. 5A, lane 9). After induction with AHT, CusS was found in the membrane fraction and additionally in the cellular debris, indicating that some CusS may have ended up in inclusion bodies under these conditions. CusS was produced in E. coli in *trans* from a plasmid as a *Strep*-tagged protein and was located in the membrane. Expression of *cusS* from this plasmid resulted in a complete restoration of copper resistance in the Δ*cusS*-mutant strain under anaerobic conditions (Fig. 6), while the copper resistance of the Δ*cusS* mutant with only the vector without gene insert remained at a low level. It can be concluded, therefore, that the *Strep*-tagged version of CusS produced from a plasmid was fully functional.

### The periplasmic region of CusS is required for copper resistance.

Because Cu(I) may bind to several sites in the periplasmic region of CusS (24), mutations in single-amino-acid residues might possibly have no effect on CusS function. Therefore, a larger portion of the periplasmic domain of CusS was removed by introducing a corresponding deletion in the coding region of the *cusS* gene (Fig. 2). The coding sequence of a disordered loop in the periplasmic portion of CusS was removed. Deletions did not affect the sequences encoding the two alpha-helices, which continue into the transmembrane alpha-helices and contain the Cu(I)-binding site H176, F43, and H42 (Fig. 2, red). Such a deletion would most likely disturb the overall structure of CusS. Instead, two amino acid sequences with metal-binding motifs were removed, specifically those in the histidine-rich stretch at positions 136 to 148 with H138, H149, H142, M143, and H145 (Fig. 2, blue and green) and at positions 129 to 138 with the methionine triad M133, M134, and M135 (magenta and blue). Moreover, a peptide spanning both regions (positions 129 to 153) was also removed. An even larger deletion (positions 67 to 153; Fig. 2, in yellow) should disturb the structure of CusS and served as a negative control.

The CusSΔ(67 to 153) derivative with the largest deletion was produced in E. coli but was apparently degraded when the expression of the corresponding gene was induced by addition of 200 μg/L AHT instead of 10 μg/L (Fig. 5B, lanes 2 and 3). The protein was located in the cell debris and in the membrane fraction. The mutant proteins CusSΔ(129 to 152), CusSΔ(136 to 148), and CusSΔ(129 to 138) were identified in the crude extract, were enriched in the debris, and were more abundant in the membrane fraction, but they were not identified in the soluble fraction (Fig. 5C and D). Thus, all four deletion derivatives of CusS were produced in E. coli and were located in the membrane fraction.

The CusSΔ (129 to 138) derivative, which no longer contained the histidine-rich stretch, mediated copper resistance on the level of the full-length protein (Fig. 6, open inverted triangles). Resistance mediated by CusSΔ(136 to 148) without the methionine triad was lower (open triangles) than copper resistance in the strain complemented with full-length CusS but was much higher than that of the strain with the vector control. When both regions were deleted, the mutant protein could no longer mediate upregulation of *cus* (open squares). Copper resistance was similar to that of the vector control (open circles) and to that mediated by the CusS derivative with the largest deletion CusSΔ(67 to 153) (open diamonds).

Together, these data indicate that CusS has a periplasmic domain that is required for the copper-mediated function of the histidine kinase.

## DISCUSSION

The CusRS system from E. coli was used to verify that the signals sensed by the histidine kinase CusS originate from the periplasm. PhoA and LacZ protein fusions defined the topology of membrane-bound CusS and clearly demonstrated that a domain of CusS that was predicted to be in the periplasm indeed was located in this compartment. In agreement with published data (3), a copper-dependent activation of CusS was required for *cusCFBA* expression and consequently Cus-mediated copper resistance under anoxic conditions (Fig. 3 and 4). Cus was required under these conditions (5) because the periplasmic Cu(I) oxidase CueO could not function due to the absence of its electron acceptor, molecular oxygen.

The residues T17, F18, and V202 within the two transmembrane alpha-helices (Fig. 1) are in close proximity to each other in the active CzcS dimer. Distance changes from intramolecular in the signaling-inactive CusS dimer form to intermolecular in its active form (28). Both transmembrane alpha-helices continue into alpha-helices that define the beginning and the end of the metal-sensing periplasmic domain of CusS. The alpha-helices downstream of M38 and upstream of N185 (Fig. 2), including the Cu(I)-binding site H42, F43, and H176 (24), delimit the beginning and the end of the periplasmic part of CusS (Table 1). Disturbance of the conformation downstream of F43 and upstream of H176 affected the functionality of CusS. In particular, the flexible loop from S129 to N152 was important for function of the protein. Loss of the histidine-rich loop (Fig. 2, blue and green) could be fully substituted by binding of Cu(I) to other sites, loss of the methionine triad (magenta) to a large degree (Fig. 6).

Binding of substrate ions to the residues F43, H42, and H176 (Fig. 2), which are located in the periplasmic part of both alpha-helices close to the outer face of the cytoplasmic membrane, triggers change of the conformation of the CusS dimer (8, 24). Studies conducted with the H34, H38, and H171 amino acid residues in CorS of Myxococcus xanthus confirm this hypothesis for CusS (29). Additional metal-binding sites in a loosely organized loop of the periplasmic domain are involved in activation of CusS (Fig. 6). Methionine-rich loops, such as those formed by M133, M134, and M135, are also involved in binding of copper to blue copper-dependent oxidases (30). Together, these data demonstrate that periplasmic copper ions are the signals that are sensed by CusS.

CusS and related transition metal-sensing HKs plus their associated response regulators use periplasmic cations as signals to control the expression of their genes. This provides further evidence that transenvelope efflux systems, which are encoded by determinants together with the genes for a two-component regulatory system, function to remove periplasmic metal cations from the periplasm before these cations can be imported into the cytoplasm. This ultimately results in a decrease of the cytoplasmic concentration of the respective metal. In this way, the transenvelope system also interacts with efflux systems of the inner membrane, which antagonize the action of the uptake systems. The cell thus achieves optimal control over the cytoplasmic metal ion concentration by indirectly restricting the import/export by the inner membrane system. Moreover, the cations are exported in a two-step mechanism, first by the inner membrane efflux system to the periplasm and later by the transenvelope efflux system. Demonstration of the dependence of CzcCBA-mediated Co(II) resistance in *C. metallidurans* on the presence of DmeF clearly supports this hypothesis (31).

In M. xanthus, the CusRS homologs CorRS also control expression of a gene for a copper-exporting P-type ATPase (29). Consequently, the genome of M. xanthus contains genes for orthologs of CusA, for instance WP_011551109. In *C. metallidurans* and Pseudomonas syringae, CusRS orthologs control the expression of genes for a CueO-like periplasmic Cu(I) oxidase plus helper genes (32–36). In contrast, *cueO* expression in E. coli is not under the control of a two-component regulatory system but instead is controlled by the MerR-type regulator CueR, which also controls *copA* expression (18–20, 37–39). Under aerobic conditions, the number of *copA* and *cueO* mRNAs reach a peak level of about 350 and 180 copies per cell, respectively, when E. coli is challenged by 1 mM Cu(II). Subsequently, the number of mRNA copies of either gene decreases to about 20 copies per cell within 20 min (40). Under anaerobic conditions, these numbers reach 1,200 copies per cell for *copA* and about 350 copies per cell for *cueO* within 20 min and remain at this level subsequently if the anaerobic conditions persist. Because CueR senses Cu(I) in the cytoplasm (38), this indicates a rapid increase in cytoplasmic Cu(I) after the cells are confronted with 1 mM Cu(II). Accumulation of cytoplasmic Cu(I) could be decreased again by the action of CueO and CopA under aerobic but not anaerobic conditions, indicating that oxidation of periplasmic Cu(I) by CueO results in decreased cytoplasmic Cu(I) accumulation; Cu(I) is a better substrate for import than Cu(II).

Evidence presented in this publication strengthens the hypothesis that the CusRS system senses periplasmic Cu(I) to control the expression of *cus*. E. coli as a facultative anaerobic bacterium can be confronted with a higher ratio of Cu(I) to Cu(II) than an aerobic bacterium such as *C. metallidurans*. E. coli suffers from a high probability that periplasmic Cu(I) oxidation by CueO may not function, making CueO a facilitator for CopA, as is evident from their common regulation by the MerR-type one-component regulator CueO.

## MATERIALS AND METHODS

### Bacterial strains and growth conditions.

E. coli strains and primers are provided in Tables 2 and 3. Tris-buffered mineral salts medium (41) containing 2 g/L sodium gluconate (TMM) was used. E. coli was cultivated under aerobic conditions at 37°C with 2 g/L glycerol and under anaerobic conditions in Hungate tubes with 2 g/L glucose instead of gluconate in a TMM adapted for E. coli. LB medium was also used (Becton, Dickinson, Heidelberg, Germany) (42, 43).

**TABLE 2:** Strains

Strain	Relevant markers	Reference
	Escherichia coli	
W3110	Wild type (K-12 derivative)	56
ECA718	Δ*cusS*	This study
ECA721	Δ*copA* Δ*cueO*	This study
ECA722	Δ*copA* Δ*cueO* Δ*cusS*	This study
ECA729	Δ*barA* Δ*uhpB* Δ*yedV*	This study
ECA731	Δ*barA* Δ*cusS* Δ*uhpB* Δ*yedV*	This study
ECA732 pECD1169	*cusS-Strep*-tag II (N-terminal) in pASK-IBA7	This study
ECA733 pECD1170	*cusS*Δ(E67 to L153)-*Strep*-tag II (N-terminal) in pASK-IBA7	This study
ECA734 pECD1171	*cusS*Δ(S129 to N152)-*Strep*-tag II (N-terminal) in pASK-IBA7	This study
ECA735 pECD1172	*cusS*Δ(S129 to H138)-*Strep*-tag II (N-terminal) in pASK-IBA7	This study
ECA736 pECD1173	*cusS*Δ(P136 to W148)-*Strep*-tag II (N-terminal) in pASK-IBA7	This study
EC771	′*blaM*′-′*phoA*; cytoplasmic PhoA control	This study
EC772	′*blaM*′-′*phoA*; periplasmic PhoA control	This study
ECA737	pECD636-c*usS*_AS15-′*lacZ*	This study
ECA738	pECD636-*cusS*_AS17-′*lacZ*	This study
ECA739	pECD636-*cusS*_AS38-′*lacZ*	This study
ECA740	pECD636-*cusS*_AS112-′*lacZ*	This study
ECA741	pECD636-*cusS*_AS185-′*lacZ*	This study
ECA743	pECD636-*cusS*_AS207-′*lacZ*	This study
ECA746	pECD636-*cusS*_AS480-′*lacZ*	This study
ECA747	pECD637-*cusS*_AS15-′*phoA*	This study
ECA748	pECD637-*cusS*_AS17-′*phoA*	This study
ECA749	pECD637-*cusS*_AS38-′*phoA*	This study
ECA750	pECD637-*cusS*_AS112-′*phoA*	This study
ECA751	pECD637-*cusS*_AS185-′*phoA*	This study
ECA753	pECD637-*cusS*_AS207-′*phoA*	This study
ECA756	pECD637-*cusS*_AS480-′*phoA*	This study

**TABLE 3:** Primers

Primer	Orientation	5′→3′sequence
*cusS* 1646 P1 up	→	GGC GTG GGT TAC ATG CTT GAG GTG CCG GAT GGT CAG TAA GCG ATT GTG TAG GCT GGA GCT
*cusS* 3117 P2 down	←	TTT TTA CAC TGG TTA TAA AAG TTG CCG TTT GCT GAA GGA CCA TGG TCC ATA TGA ATA TCC TCC
JR *barA* P1 up	→	TTT AAC AGT GTG ACC TTA ATT GTC CCA TAA CGG AAC TCC GCG ATT GTG TAG GCT GGA GCT
JR *barA* P2 down	←	GCG TCA TAA AAA GCC GAT TGC TAC TCG ACA AGA CAT CCA CCA TGG TCC ATA TGA ATA TCC TCC
JR *uhpB* P1 up	→	TAG AGC TGG CGC GCC GCA TGT TTG ATG GCT GGT GAT GAA GCG ATT GTGT TAG GCT GGA GCT
JR *uhpB* P2 down	←	ATC GGC AGG CGC TTT CAG AAA CGG CAA CAT CAT CAA ACC CCA TGG TCC ATA TGA ATA TCC TCC
JR *yedV* P1 up	→	CCG GGG GAT GGG CTA TTC ATT CGT AGC GGT AAA AAA ATA GCG ATT GTG TAG GCT GGA GCT
JR *yedV* P2 down	←	TTT TTT CAC GGT TAA TTT ATG GCG TAC TGA AGC CCT ATG CCA TGG TCC ATA TGA ATA TCC TCC
CusSEco1004uIBA3	→	AAA GAA TTC GTC AGT AAG CCA TTT CAG CGC C
CusSNco2417dIBA3	←	AAA CCA TGG AGC GGG TAA TGT GAT AAC AAA CCT
CusS mut SacI 1193 d	←	AAA GAG CTC GTC AGG GTG ATT TAG TAC CCG
CusS mut SacI 1460 u	→	AAA GAG CTC CCG GTT GGC CCG TTG G
CusS mut SacI 1387 d	←	AAA GAG CTC GGA AAG GAG ATA CAC CTC GCC
CusS mut SacI 1454 u	→	AAA GAG CTC AAC TTG CCG GTT GGC CC
CusS mut SacI 1412 u	→	AAA GAG CTC CAC GGT CAC GGG CAT ATG G
CusS mut SacI 1408 d	←	AAA GAG CTC TGG CAT CAT CAT CGT CGG G
CusS mut SacI 1442 u	→	AAA GAG CTC TGG CGG ATG ATT AAC TTG CCG
*phoA cusS* 991 Kpn u	→	AAA GGT ACC GGA GGA GCC GGA TGG TCA GTA
AS15 *cusS* 1045 Xba	←	AAA TCT AGA GCG GGT TGC CAG CGA AAA
AS17 *cusS* 1051 Xba	←	AAA TCT AGA GGT CAG GCG GGT TGC CAG
AS38 *cusS* 1114 Xba	←	AAA TCT AGA TGA GTG GAT CAT GAT CCA TGC AA
AS112 *cusS* 1336 Xba	←	AAA TCT AGA ACG CGT AAA CTC GCG GAT ATC
AS185 *cusS* 1558 Xba	←	AAA TCT AGA TTT ATT CAT CAA ATC ATT TAT GTA ATG
AS207 *cusS* 1624 Xba	←	AAA TCT AGA TTT ATG TAC CGC CAA CAG TAC GAT AA
AS480 *cusS* 2441 Xba	←	AAA TCT AGA AGC GGG TAA TGT GAT AAC AAA CCT

Dose-response growth curves were conducted in TMM or LB. A preculture was incubated for 16 h at 37°C and 200 rpm up to early stationary phase, diluted 1:400 into fresh medium with glycerol as a carbon source in the case of TMM, incubated for 24 h at 37°C and 200 rpm, diluted again 400-fold into fresh medium with glucose as a carbon source, and incubated for 2 h with shaking at 37°C. CuCl_2_ was added, the incubation was continued for 6 h at 37°C, and the turbidity was determined at 600 nm using a Spectronic 20+ (Milton Roy, Ivyland, PA). Under anaerobic conditions, TMM contained 2 g/L glucose, and the incubation time with copper ions was 16 h; LB contained 0.5 g/L glucose, and the incubation time was 6 h before the turbidity was measured.

### Genetic techniques.

Standard molecular genetics techniques were used (44, 45). Genes were deleted by insertion of resistance cassettes using the λ Red-recombinase system (46), as previously published (21). Initial deletions were performed in E. coli strain BW25113, in which the target genes were exchanged for a chloramphenicol (*cat*) resistance cassette, and subsequently transferred by general transduction with phage P1 into E. coli strain W3110 or its derivatives. In the resulting mutant strains, the genes were disrupted by insertion of the *cat* resistance cassette through homologous recombination. Multiple deletions were constructed by FLP recombination target (FRT)-dependent elimination of the respective resistance cassette assisted by flippase from plasmid pCP20 (46) and subsequent general phage P1 transduction. All mutations were verified by PCR.

### Reporter gene fusions.

Fusion vectors pECD636 (*lacZ* fusions) and pECD637 (*phoA* fusions) (12) were used. These are derivatives of plasmids pECD499 and pECD500 (47), respectively; however, the translational fusion proteins were expressed under the control of the phage T7 promoter (48) in E. coli CC118(pGP1-2) (26, 48). The specific activities of alkaline phosphatase (26) and beta-galactosidase (49) were determined at least in triplicate, as published previously (50). The fusion proteins produced in these experiments were analyzed as previously described (48, 51); however, the polypeptides were not labeled with [^35^S]methionine. Instead, after separation by SDS-PAGE (52), the proteins were blotted onto a polyvinylidene difluoride (PVDF) membrane (Roche, Mannheim) and visualized with an anti-LacZ or anti-PhoA rabbit primary antibody and a secondary anti-rabbit goat peroxidase conjugate antibody, followed by chemiluminescence as described by the manufacturer (Lumi-Film, Roche, Mannheim).

### CusS.

The *cusS* gene and its derivatives were cloned into pASK-IBA7 (IBA, Göttingen, Germany), which attaches an amino-terminal *Strep* Tag II under the control of the *tet* promoter in E. coli strain BL21(pLys). Precultures in LB were diluted 50-fold into fresh LB and were incubated with shaking at 37°C until the turbidity at 600 nm reached 1.0. Anhydrotetracycline (AHT) was added to a final concentration of 10 or 200 μg/L. Incubation was continued with shaking at 30°C, and the cells were harvested by centrifugation. Crude extract was prepared by ultrasonication followed by removal of the debris by centrifugation for 30 min at 4°C and 5,000 rpm (Eppendorf 5804R, Eppendorf, Hamburg). The crude extract was further separated into membrane and soluble fractions by ultracentrifugation (1 h at 45,000 rpm and 4°C; Beckman L8-M ultracentrifuge). Following SDS-PAGE (52) of samples representing 50 μg of dry mass or 25 μg of protein in a linear 10% (wt/vol) gel, the proteins were transferred by semidry electroblotting onto a nitrocellulose membrane (NCP, Macherey-Nagel, Düren, Germany) at 3 mA/cm^2^ and 15 V for 30 min and detected using a *Strep*-Tactin peroxidase conjugate antibody (Institut für Bioanalytik Göttingen, Germany) and chemiluminescence detection, as described by the manufacturer (Lumi-Film, Roche, Mannheim, Germany).

### Statistics.

Student’s *t* tests were used, but, in most cases, the distance (*D*) value has been used several times previously for such analyses (53–55). It is a simple, more useful value than Student’s *t* tests because nonintersecting deviation bars of two values (*D* > 1) for three repeats always means a statistically relevant (≥95%) difference provided that the deviations are within a similar range. At *n* = 4, significance is ≥97.5%, at *n* = 5, significance is ≥99% (significant), and at *n* = 8, significance is ≥99.9% (highly significant).
